# *Enterocytozoon bieneusi* infection disrupts bile acid metabolism in the wild rodent gut microbiota: adaptive shifts in microbial metabolism and community structure

**DOI:** 10.3389/fcimb.2025.1647377

**Published:** 2025-09-22

**Authors:** Kai-Meng Shang, He Ma, Yong-Jie Wei, Ji-Xin Zhao, Ya Qin, Jian-Ming Li, Zi-Yu Zhao, Hai-Long Yu, Quan Zhao, Bei-Ni Chen, Hany M. Elsheikha, Xiao-Xuan Zhang, Xing Yang

**Affiliations:** ^1^ Integrated Laboratory of Pathogenic Biology, College of Preclinical Medicine, Dali University, Dali, China; ^2^ College of Life Sciences, Changchun Sci-Tech University, Shuangyang, Jilin, China; ^3^ College of Veterinary Medicine, Qingdao Agricultural University, Qingdao, Shandong, China; ^4^ College of Veterinary Medicine, Jilin Agricultural University, Changchun, Jilin, China; ^5^ Faculty of Medicine and Health Sciences, School of Veterinary Medicine and Science, University of Nottingham, Loughborough, United Kingdom

**Keywords:** gut microbiota, microbial functional profiling, bile acid metabolism, *Enterocytozoon bieneusi*, rodentia

## Abstract

**Introduction:**

Bile acids (BAs) are central to host–microbiota interactions, yet their metabolism in wild rodents remains poorly characterized. This study aimed to explore the genomic potential of gut microorganisms in wild rodents for BA metabolism and its implications for host adaptation and pathogen interactions.

**Methods:**

We reconstructed 6,332 genomes from the gut microbiota of wild rodents and performed genome-resolved metabolic profiling. Comparative analyses were conducted across host species, including humans, pigs, laboratory mice, and chickens. Functional enrichment was further assessed in relation to glycoside hydrolase families and Enterocytozoon bieneusi infection status.

**Results:**

A total of 5,208 genomes were identified as participants in key BA metabolic pathways, including deconjugation, oxidation, and dihydroxylation, predominantly from Bacillota_A and Bacteroidota. Notably, Muribaculaceae and CAG-485 lineages within Bacteroidota encoded bile salt hydrolase (BSH). Cross-species comparisons revealed a striking absence of 7β-hydroxysteroid dehydrogenase (7β-HSDH) in laboratory mice, indicating their limited suitability for modeling intestinal BA metabolism. BSH-encoding genomes were significantly enriched in glycoside hydrolase families GH13 and GH16, suggesting a potential link between BA transformation and carbohydrate metabolism. Furthermore, Enterocytozoon bieneusi infection was associated with a marked increase in BA-related microbial taxa in wild rodents.

**Discussion:**

Our findings highlight the intricate interconnections between gut microbial functions, BA metabolism, and pathogen interactions. The absence of 7β-HSDH in laboratory mice underscores wild rodents as potentially more suitable models for BA research. These results open new avenues for understanding microbiome-driven host adaptation and health.

## 1 Introduction

The gut microbiota plays a central role in host metabolism, influencing digestion, immune function, and susceptibility to disease (Krishnan et al., 2015). Among the many microbial processes within the gut, bile acid (BA) metabolism is particularly significant due to its involvement in lipid absorption, intestinal homeostasis, and the regulation of metabolic disorders (Guan et al., 2022; Wang et al., 2024b). BAs, synthesized in the liver, undergo extensive microbial modifications in the gut, which not only modulate their chemical properties but also reshape host metabolic pathways (Ramírez-Pérez et al., 2017).

While BA metabolism has been extensively studied in model organisms (Staley et al., 2017; Mohanty et al., 2024), much less is known about these microbial functions in wild animals. In particular, the gut microbiomes of wild rodents, an ecologically diverse and abundant group, remain underexplored. These animals offer a unique opportunity to study microbiota-driven metabolic processes in natural settings (Rosshart et al., 2019). Unlike their laboratory counterparts, wild rodents are exposed to variable diets, environments, and microbial landscapes (Rosshart et al., 2017; Schmidt et al., 2019), all of which can shape distinct microbiome compositions and functional capacities, including BA metabolism. Bridging this gap is essential for advancing our understanding of microbiome diversity and its influence on host physiology beyond controlled experimental systems.

Moreover, the gut microbiota is highly responsive to pathogenic infections, which can disrupt microbial balance and alter host metabolism (Vich Vila et al., 2018; Trzebny et al., 2023). In this context, it is important to note that laboratory mice maintained under controlled, pathogen-free conditions exhibit markedly different immune responses from their wild counterparts (Rosshart et al., 2017; Beura et al., 2016; Mair et al., 2021), largely because the latter inhabit complex natural environments where coinfections are common and many sources of variation present in nature are eliminated in the laboratory. Among the pathogens relevant to such natural settings, one such notable example is *Enterocytozoon bieneusi*, a microsporidian parasite, has been shown to significantly reshape gut microbial communities with potential consequences for host health (López-Carvallo et al., 2022). However, the effects of *E. bieneusi*-induced microbiota shift on BA metabolism remain largely uncharacterized, especially in wild rodent populations. Investigating these dynamics may uncover novel interactions between pathogens, microbiota, and host metabolic pathways.

In this study, we performed a genome-resolved metagenomic analysis of the gut microbiota in wild rodents, with a specific focus on microbial taxa and functional genes involved in bile acid metabolism. Leveraging a high-quality genomic dataset, we characterized microbial diversity, taxonomic structure, and key BA-related pathways, while also assessing the impact of *E. bieneusi* infection on these microbial communities. Our findings provide new insights into the ecological and metabolic roles of the wild rodent gut microbiome and highlight the complex interplay between host, microbiota, and pathogen in shaping metabolic function and health outcomes.

## 2 Materials and methods

### 2.1 Data collection, genome preprocessing and gene prediction

We assembled a comprehensive dataset comprising 14,061 gut microbiome genomes from wild rodents, generated by our laboratory (Shang et al., 2025). To ensure data quality and reliability, genome completeness and contamination were evaluated using CheckM2 (v1.0.1) (Chklovski et al., 2023). To ensure stringent quality standards, we adopted more rigorous thresholds than those used in previous studies (Lin et al., 2023), retaining only genomes with ≥ 80% completeness and ≤ 5% contamination for downstream analyses.

To remove redundancy, genome dereplication was performed using dRep (v3.4.3) (Olm et al., 2017). We applied distinct similarity thresholds depending on resolution: for strain-level dereplication, parameters were set at -pa 0.9, -sa 0.99, -nc 0.30 and for species-level clustering, we used -pa 0.9, -sa 0.95, -nc 0.30. High-quality, non-redundant genomes were taxonomically classified using the classify_wf module of GTDB-Tk (v2.3.2) (Chaumeil et al., 2022), referencing the GTDB database for consistent phylogenomic placement. Open reading frames (ORFs) were predicted for each genome using Prodigal (v2.6.3) (Hyatt et al., 2010), enabling subsequent functional annotation.

To explore phylogenetic relationships, we constructed a maximum likelihood tree using PhyloPhlAn (v3.0.67) (Asnicar et al., 2020). Tree visualization and annotation were performed using the iTOL (v6.9.1) (Letunic and Bork, 2021), allowing clear representation of taxonomic structure and genome-level traits. To assess the relative abundance of strain-level genomes, we analyzed metagenomic reads derived from our previous study of *E. bieneusi* infection (BioProject: PRJNA1175865). Clean reads from infected (*n* = 10) and uninfected control (*n* = 10) wild rodents were aligned to the dereplicated genome set using Bowtie2 (v2.5.0) with default parameters (Langmead and Salzberg, 2012). Read counts were normalized to transcripts per kilobase million (TPM) to facilitate accurate comparisons of genome abundance across samples.

### 2.2 Functional annotation

To characterize the functional potential of the gut microbiome, protein-coding sequences were annotated using DIAMOND (v2.1.8.162) (Buchfink et al., 2015) against the Kyoto Encyclopedia of Genes and Genomes (KEGG) database. Searches were conducted with the parameters –min-score 60 –query-cover 70 to ensure high-confidence matches. From the resulting KEGG orthologs (KOs), we specifically extracted those associated with secondary bile acid biosynthesis (KEGG pathway map00121), including the following key KOs: K00076, K01442, K07007, K15868–K15874, K22604–K22607, and K23231. The presence, genomic location, and copy number of these KOs were determined for each genome. To further evaluate carbohydrate metabolism potential, protein-coding genes were annotated using the Carbohydrate-Active enZYmes (CAZy) database (Lombard et al., 2014). DIAMOND searches were performed with the parameters –min-score 60 and –query-cover 50. For both KEGG and CAZy annotations, the alignment with the highest bit score was selected as the best hit and used to assign functional and taxonomic identities to the corresponding ORFs.

### 2.3 Statistical analyses and visualization

All statistical analyses were performed in R (v4.2.2). Microbial taxonomic and functional gene abundance data were used to calculate alpha diversity metrics, including Richness and Shannon indices. β-diversity was assessed using Principal Coordinate Analysis (PCoA) based on Bray-Curtis dissimilarity, with group differences evaluated using permutational multivariate analysis of variance (PERMANOVA). To compare diversity indices, taxonomic profiles, and functional gene abundances between groups, the Wilcoxon rank-sum test was applied. Results were considered statistically significant at *p* < 0.05, unless otherwise specified. For data visualization, heatmaps were produced using the ComplexHeatmap R package (v2.8.0), while Sankey diagrams were generated using the ‘ggsankey’ package (v0.0.9). All other visualizations, including boxplots, bar charts, and ordination plots, were generated using the ‘ggplot2’ package (v4.2.3).

## 3 Results

### 3.1 Collection, quality assessment, and taxonomic characterization of intestinal genomes from wild rodents

**Figure 1:**
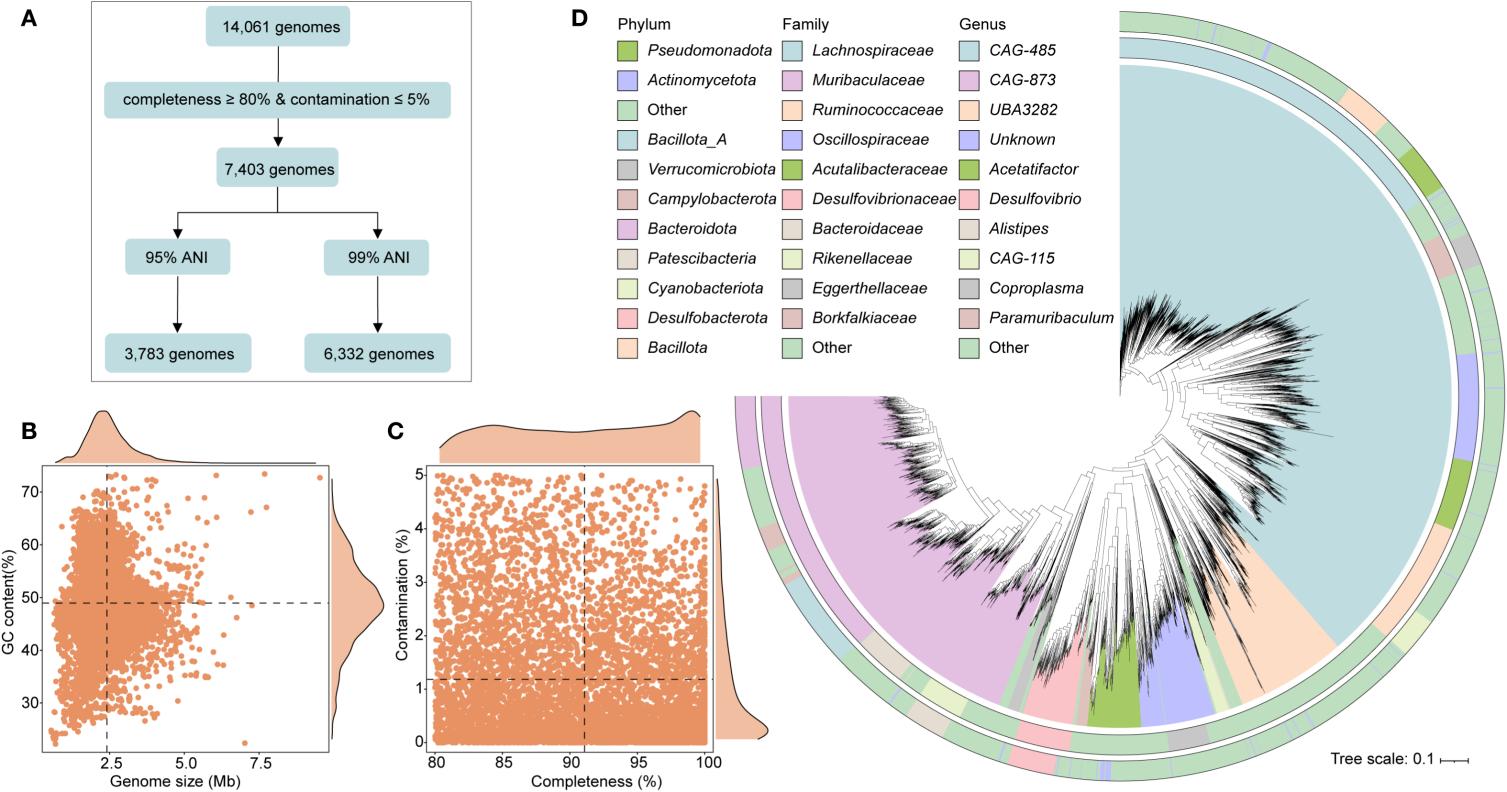
Genomic characteristics of intestinal microbiota from wild rodents. **(A)** Workflow illustrating the genome processing pipeline, including quality filtering and redundancy removal. **(B, C)** Summary statistics of the 6,332 high- and medium-quality genomes, showing genome size, GC content, completeness, and contamination. **(D)** Maximum likelihood phylogenetic tree of 3,783 species-level genome bins (SGBs), colored by phylum and annotated with dominant families.

Following quality control (completeness ≥ 80%, contamination ≤ 5%), a total of 7,403 genomes were initially recovered. After removing redundancy using a 99% average nucleotide identity (ANI), 6,332 non-redundant genomes were retained for downstream analysis (**Figure 1A**). The genomes varied in size from 0.55 to 9.54 Mbp (mean: 2.41 Mbp), with GC content ranging between 22.21% and 73.42% (mean: 48.96%) (**Figure 1B**). On average, genome completeness was 91.08%, while contamination remained low at 1.18% (**Figure 1C**). Among the retained genomes, 3,507 genomes (28.74%) met high-quality standards (completeness ≥ 90% and contamination ≤ 5% (**Supplementary Table 1**). Gene prediction across the dataset yielded a comprehensive catalog of 14,030,587 genes. Species-level genome bins (SGBs) were defined by clustering genomes at 95% ANI, resulting in 3,783 unique SGBs. Taxonomic classification revealed broad microbial diversity, encompassing 23 phyla, 156 families, and 621 genera. The majority of SGBs belonged to the phylum *Bacillota_A* (50.78%), followed by *Bacteroidota* (25.03%) and *Bacillota* (7.14%). At the family level, the most abundant were *Lachnospiraceae* (20.86%), *Muribaculaceae* (15.89%), and *Ruminococcaceae* (7.38%) (**Figure 1D**; **Supplementary Table 1**).

### 3.2 Genomic characterization of bile acid transformation pathways in the intestinal microbiota of wild rodents

**Figure 2:**
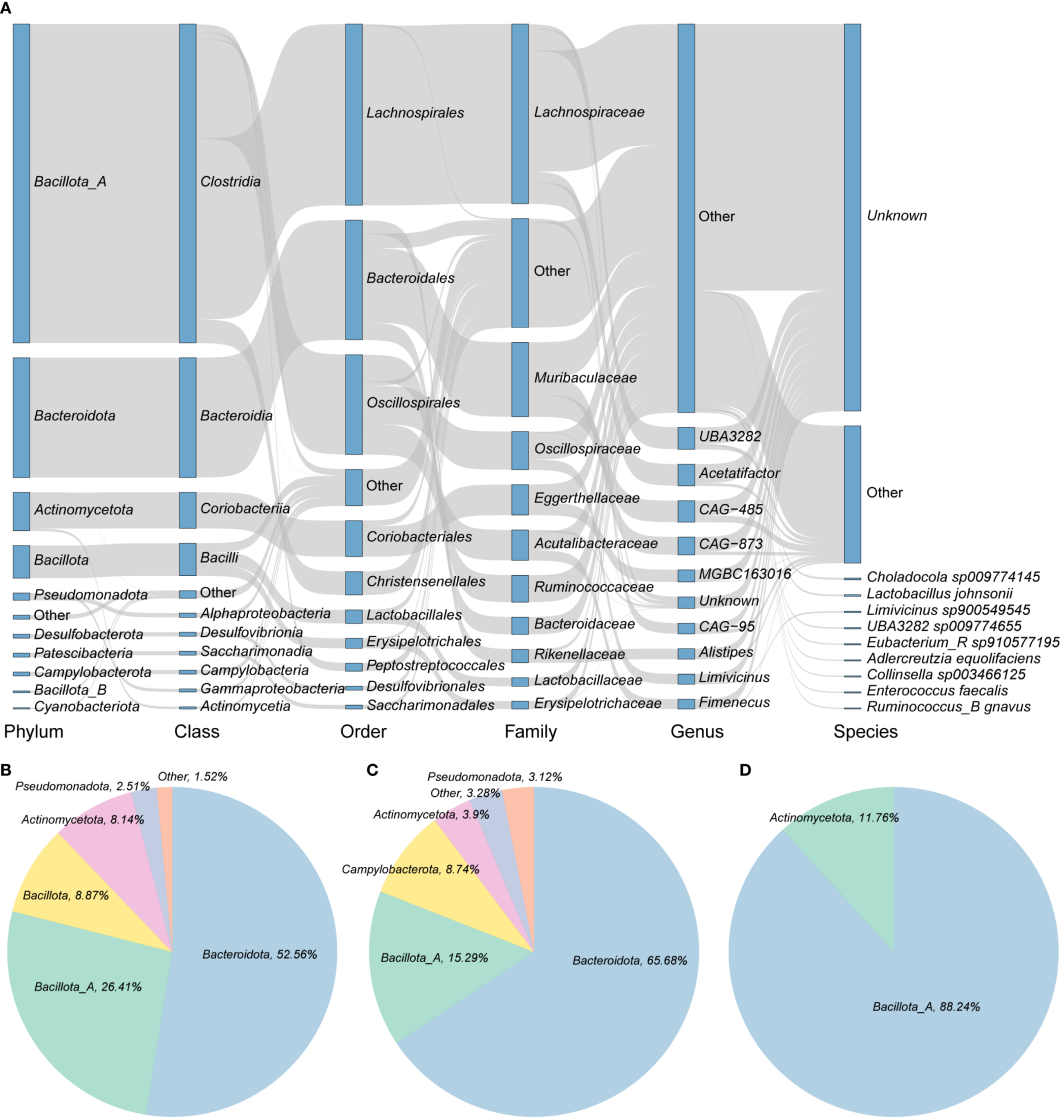
Bile acid transformation capacity of intestinal microbiota in wild rodents. **(A)** Taxonomic distribution of the 5,208 genomes carrying bile acid (BA) transformation genes. Rectangles represent taxonomic levels; their lengths correspond to the number of genomes in each group. **(B–D)** Proportions of genomes encoding key enzymes involved in BA metabolism: **(B)** BSH, **(C)** 7α-HSDH, and **(D)** baiB.

This study investigated the potential of gut microbiota in wild rodents to mediate BA transformation through KEGG-based functional annotation. A total of 10,051 genes associated with BA metabolic pathways, specifically deconjugation, oxidation, and dihydroxylation, were identified across 5,208 genomes, representing more than 80% of the total dataset (**Supplementary Figure 1**; **Supplementary Table 2**). The majority of these genes were found in bacteria from the phylum *Bacillota_A* (primarily class *Clostridia*), followed by *Bacteroidota* and *Actinomycetota*. At the family level, *Lachnospiraceae* was the most abundant, followed by *Muribaculaceae* and *Oscillospiraceae* (**Figure 2A**). Among these 5,208 genomes, 2,818 encoded bile salt hydrolase (BSH) (choloylglycine hydrolase [K01442; EC:3.5.1.24]), an enzyme that catalyzes the deconjugation of bile salts. These BSH-carrying genomes spanned 10 phyla, with the largest contributions from *Bacteroidota* (52.56%), followed by *Bacillota_A* (26.41%) and *Bacillota* (8.87%) (**Figure 2B**). The most enriched BSH-associated families included *Muribaculaceae* (*n* = 1,064), *Lachnospiraceae* (*n* = 478), and *Rikenellaceae* (*n* = 219), with the predominant genera being *CAG-485* (*n* = 302) and *CAG-873* (*n* = 245) (**Supplementary Table 2**). Additionally, 609 genomes encoded 7-alpha-hydroxysteroid dehydrogenase (7α-HSDH) [K00076; EC:1.1.1.159]), which catalyzes the NAD(P)+-dependent oxidation of hydroxyl groups in deconjugated bile acids. These genomes were predominantly affiliated with *Bacteroidota* (65.68%), followed by *Bacillota_*A (15.29%) and *Campylobacterota* (8.74%) (**Figure 2C**). In contrast, only 34 genomes were found to encode *baiB* (bile acid–CoA ligase [K15868; EC:6.2.1.7]), a key enzyme in the 7α-dehydroxylation pathway responsible for converting primary BAs into secondary BAs via CoA ligation. These genomes belonged to just two phyla—*Bacillota_A* (88.24%) and *Actinomycetota* (11.76%) (**Figure 2D**). This limited distribution suggests that only a small subset of bacterial taxa in wild rodents possess the capacity for full secondary BA biosynthesis, highlighting a niche functional specialization.

### 3.3 Distinct bile acid transformation pathways in the intestinal microbiota of wild rodents

**Figure 3:**
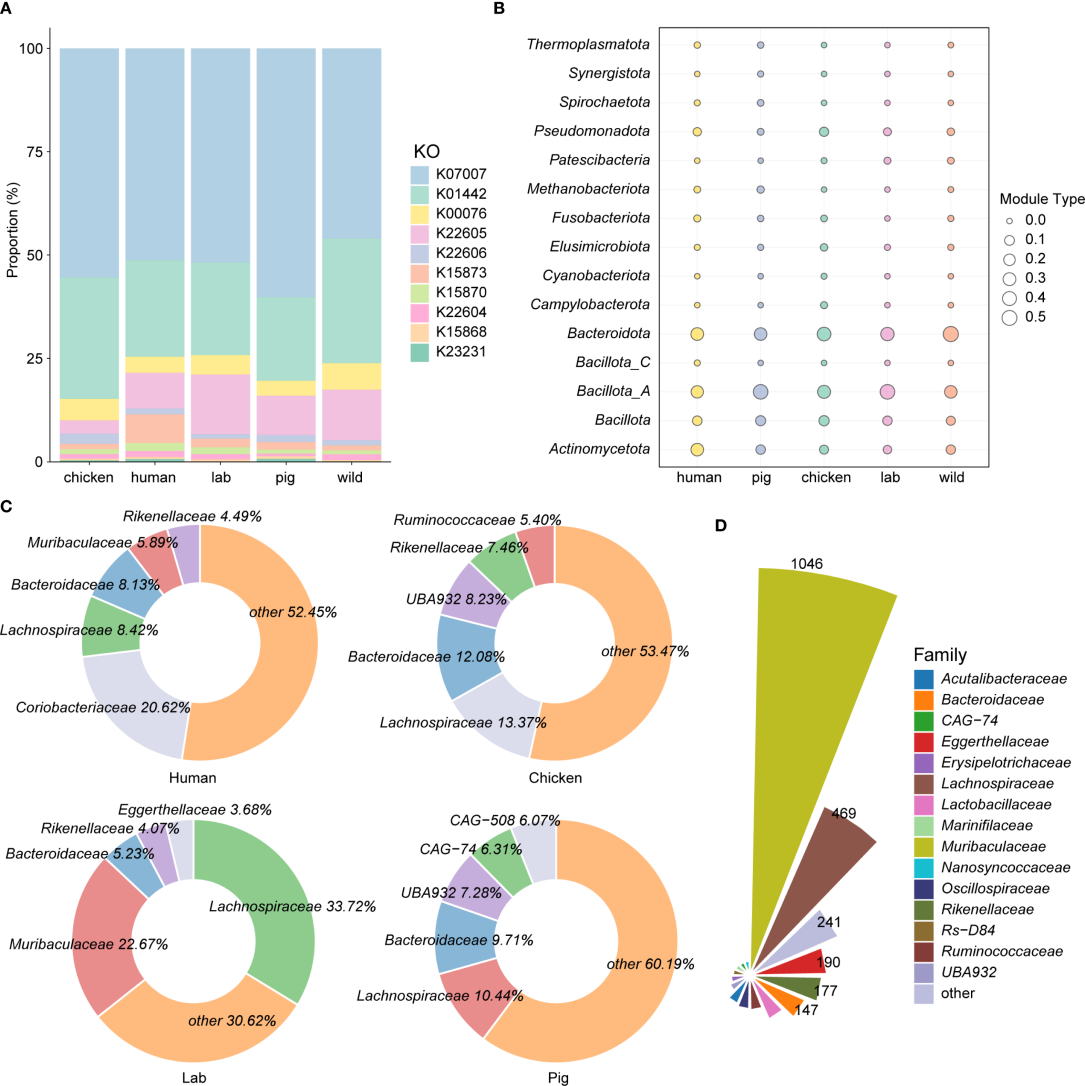
Host-specific bile acid metabolism by the intestinal microbiota. **(A)** Comparative analysis of KEGG orthologs (KOs) associated with bile acid transformation pathways across the intestinal microbiota of wild rodents, laboratory mice (Lab), humans, and pigs. **(B, C)** Taxonomic distribution of BSH-encoding metagenome-assembled genomes (MAGs) at the phylum and family levels, respectively, across different host species. **(D)** Taxonomic classification of BSH-carrying MAGs at the family level in the intestines of wild rodents.

To investigate host-specific variation in BA-metabolizing microbiota, 5,208 intestinal genomes derived from wild rodents were compared against published metagenome-assembled genomes (MAGs) from other host species, including humans (2,294 MAGs) (Nayfach et al., 2019), pigs (1,411 MAGs) (Gaio et al., 2021), chickens (2,113 MAGs), and laboratory mice (1,416 MAGs) (Kieser et al., 2022) (**Supplementary Table 3**). Functional annotation revealed that these MAGs encoded a diverse repertoire of BA-related KOs: 3,499 KOs in humans, 2,229 in pigs, 2,897 in chickens, and 2,644 in laboratory mice. Remarkably, 7β-HSDH (K23231) was absent in the laboratory mouse dataset, although it was detected in all other host species (**Figure 3A**; **Supplementary Table 3**). Focusing on BA deconjugation, BSH was widely distributed across all host species, occurring in 31.08% of human MAGs, 29.20% of pig MAGs, 36.82% of chicken MAGs, 36.44% of laboratory mouse MAGs, and a markedly higher 44.50% of wild rodent genomes.

Taxonomic profiling showed that *Bacillota_A* dominated among BSH-positive MAGs in laboratory mice and pigs, whereas *Bacteroidota* was the predominant phylum in humans, chickens, and wild rodents (**Figure 3B**). At the family level, BSH-carrying MAGs were most frequently assigned to *Lachnospiraceae* in pigs (10.44%), laboratory mice (33.72%), and chickens (13.37%), while in humans, *Coriobacteriaceae* was the most dominant family (20.62%). In contrast, wild rodents displayed a unique profile, with *Muribaculaceae* (37.20%) as the leading BSH-harboring family, followed closely by *Lachnospiraceae* (37.12%) (**Figures 3C, D**). Interestingly, the genus *CAG-485* represented the most abundant BSH carrier in wild rodents (10.56%), while its prevalence was markedly lower in other host species (**Supplementary Figure 2**). Taken together, these findings highlight distinct host-specific configurations of bile acid–metabolizing microbiota. The enrichment of *Muribaculaceae* and *CAG-485* in wild rodents suggests a specialized microbial adaptation to the environmental and dietary pressures unique to their ecological niche.

### 3.4 Functional characterization of BSH-carrying microbial genomes in the intestine of wild rodents

**Figure 4:**
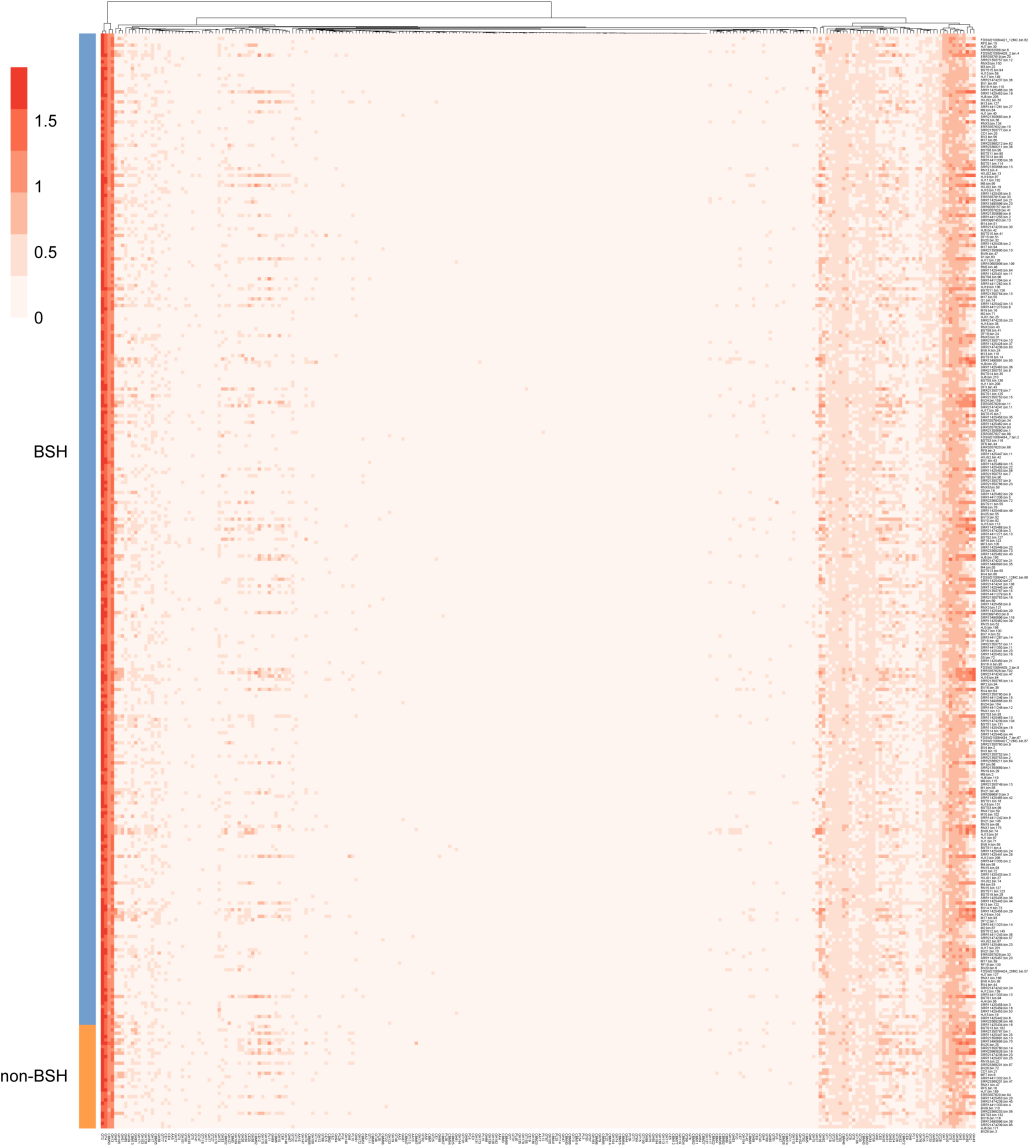
Functional enrichment of BSH-carrying genomes in the *CAG-485* genus. Functional profiling of BSH-positive and BSH-negative *CAG-485* genomes from the intestines of wild rodents. The plot highlights enrichment of specific CAZyme families, notably GH13, GH16, and GH63, suggesting their involvement in gut adaptation and potential interaction with bile acid metabolism.

The initial step in BA metabolism is the deconjugation of primary BAs, catalyzed by BSH, a critical reaction that facilitates bile tolerance and microbial adaptation to selective pressures of the intestinal environment (Guzior and Quinn, 2021; Jones et al., 2008). To investigate the functional potential of BSH-carrying microbes in wild rodents, genomes within the genus *CAG-485* were analyzed. Of the 328 genomes assigned to this genus, 31 lacked BSH and were categorized as non-BA genomes (**Supplementary Table 4**). Comparative functional profiling using carbohydrate-active enzymes revealed significant differences between BSH-positive and BSH-negative genomes. In particular, BSH-carrying genomes showed a significantly higher prevalence of GH13 and GH16, glycoside hydrolase families linked to carbohydrate metabolism and gut colonization. Strikingly, the GH63 family was found exclusively in BSH-carrying genomes and was completely absent in non-BSH genomes (**Figure 4**; **Supplementary Table 4**). Although the exact role of GH63 in BA metabolism is not yet fully defined, its strong co-occurrence with BSH suggests a synergistic functional relationship that may promote microbial fitness and resilience in the gut environment. These findings point to a distinct functional advantage of BSH-carrying microbes, highlighting their potential to coordinate BA transformation with enhanced carbohydrate metabolism, an adaptation that may confer a competitive edge within the rodent intestinal microbiome.

### 3.5 Alterations in gut microbiota and BA biosynthesis in wild rodents infected with *E. bieneusi*

**Figure 5:**
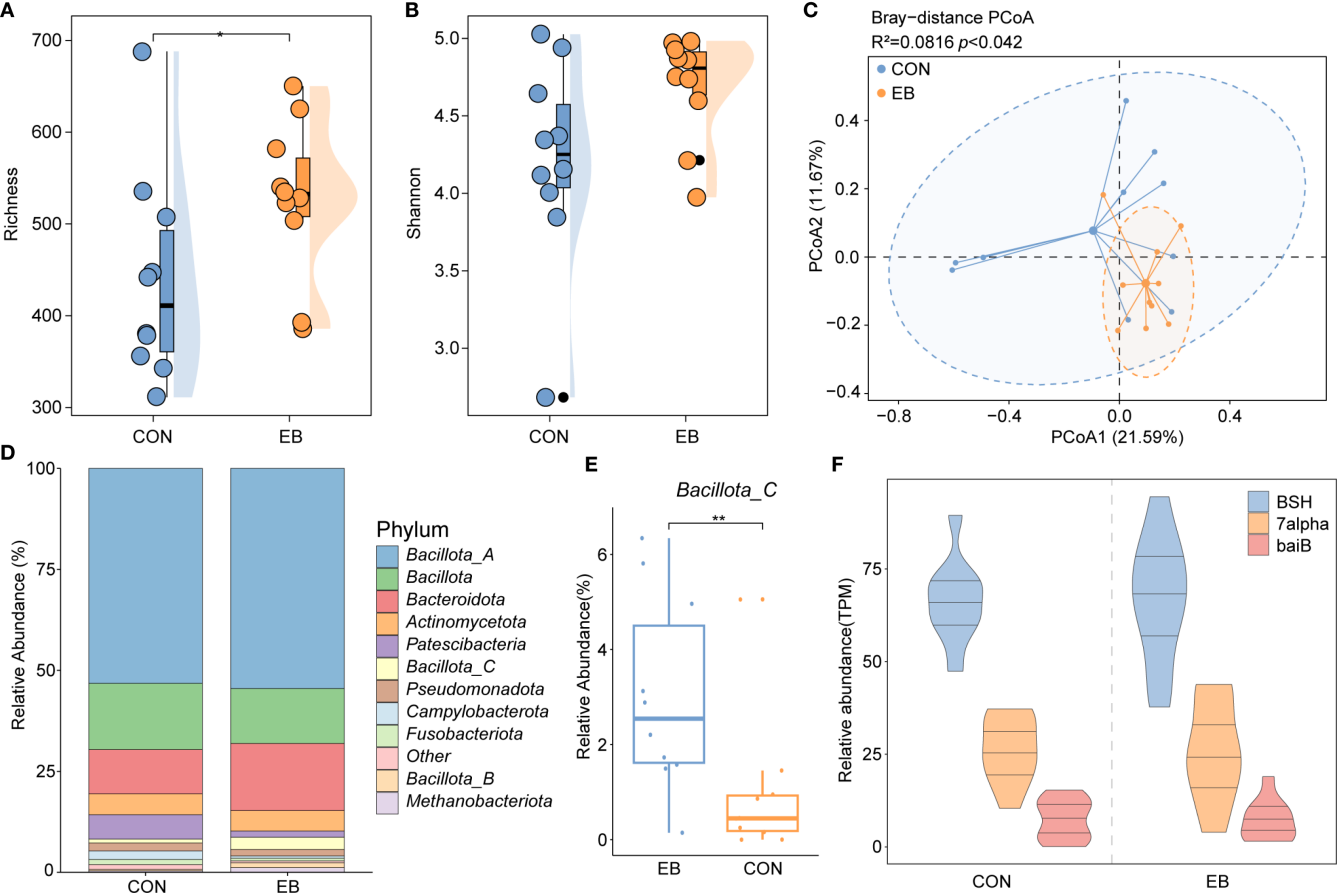
Gut microbiota and BA metabolism response to *Enterocytozoon bieneusi* infection. **(A, B)** Boxplots comparing Richness and Shannon diversity indices between the control (CON) and *E. bieneusi*-infected (EB) groups. Statistical significance was determined using the Wilcoxon rank-sum test (*, *p* < 0.05). **(C)** Principal Coordinates Analysis (PCoA) plot based on Bray–Curtis dissimilarity, illustrating β-diversity and compositional differences between groups. **(D)** Stacked bar chart showing the relative abundance of major phyla associated with BA biosynthesis in the cecal microbiota. **(E)** Boxplot comparing the abundance of phylum *Bacillota_C* between CON and EB groups (*, p < 0.05). **(F)** Relative abundance of three key bile acid metabolism genes, *BSH*, *7α-HSDH*, and *baiB*, in the CON and EB groups. **, p < 0.01.

The gut microbiota plays a crucial role in BA biosynthesis; however, its response to parasitic infection, particularly by *E. bieneusi*, remains unclear. To investigate this interaction, we analyzed the gut microbiome data from wild rodents naturally infected with *E. bieneusi*, focusing on microbial genomes involved in BA biosynthesis. Alpha and beta diversity metrics were used to assess community structure and composition. Alpha diversity, measured using the Richness and Shannon indices, revealed a significant increase in the richness of BA biosynthesis-associated genomes in the *E. bieneusi*-infected group. The Shannon diversity index was also significantly elevated compared to the control (CON) group, indicating greater community evenness and complexity post-infection (**Figures 5A, B**). PCoA based on Bray-Curtis dissimilarity demonstrated a clear shift in microbial composition between infected and uninfected groups, with a clear distinction between the *E. bieneusi* and CON groups (R² = 0.0816, *p* < 0.042), suggesting that *E. bieneusi* infection leads to significant microbiome restructuring (**Figure 5C**). Taxonomic profiling of BA biosynthesis-related genomes identified *Bacillota_A* as the most abundant phylum, followed by *Bacillota* and *Bacteroidota* (**Figure 5D**). Interestingly, *Bacillota_C* abundance was significantly higher in the *E. bieneusi*-infected group compared to the control (*p* < 0.05), highlighting a specific microbial response to infection (**Figure 5E**). Further functional analysis revealed an increased prevalence of key BA transforming enzymes, BSH, 7α-HSDH, and *baiB*, in the infected group relative to the control (**Figure 5F**). This suggest that *E. bieneusi* infection not only reshapes microbial community composition but also enhances BA biosynthesis potential, with possible implications for host metabolism and gut homeostasis.

## 4 Discussion

This study provides new insights into the gut microbiota of wild rodents, highlighting both their extensive microbial diversity and the specialized metabolic pathways involved in BA transformation. Analysis of 5,208 high-quality microbial genomes revealed distinctive compositional patterns, particularly the dominance of *Muribaculaceae*, with the genus *CAG-485* emerging as a central contributor to BA metabolism. These findings indicate that wild rodents harbor highly adapted microbial consortia capable of modulating host metabolic processes and underscore the importance of further research into the physiological consequences of microbial BA transformations.

Metagenomic sequencing enabled comprehensive functional profiling of the gut microbiome, overcoming the taxonomic and functional limitations of 16S rRNA sequencing (Claesson et al., 2010; Kennedy et al., 2014). This approach facilitated direct inference of microbial functions and enabled metagenome-wide association studies linking microbiome structure to host phenotypes and disease (Wang et al., 2018; Chaston et al., 2014). In this study, the dominant phyla were *Bacillota_A*, *Bacteroidota*, and *Actinomycetota*, with *Lachnospiraceae, Muribaculaceae*, and *Ruminococcaceae* as the most abundant families. These taxonomic patterns likely reflect adaptations to ecological niches and diet (Rinninella et al., 2019; Couch et al., 2021; Ogasawara et al., 2023).

Primary BAs are synthesized in the liver and subsequently modified gut microbiota into secondary BAs (Ridlon et al., 2006). Using metagenomic data, we identified 10,051 genes across 5,208 genomes involved in BA transformation, including deconjugation, oxidation, and dihydroxylation processes (Guzior and Quinn, 2021; Ridlon et al., 2016). The microbial community involved in BA metabolism was dominated by *Bacillota_A* (50.78%), followed by *Bacteroidota* (25.03%) and *Actinomycetota*, with key roles played by *Lachnospiraceae*, *Muribaculaceae*, and *Oscillospiraceae*. These taxa are essential to BA metabolism and may influence host immunity and inflammatory responses (Cai et al., 2022; Lloyd-Price et al., 2019).

Gut microbiota-mediated BA metabolism affects host lipid digestion, cholesterol regulation, and multiple signaling pathways (Yang et al., 2021). A pivotal reaction in this pathway is the hydrolysis of conjugated BAs, catalyzed by bile salt hydrolases (Ridlon and Gaskins, 2024). While BSH activity has traditionally been associated with *Firmicutes* (Kisiela et al., 2012), our data reveal a broader taxonomic distribution, with significant contributions from *Bacteroidota* and *Bacillota_A*. BSH genes were identified in genera including *Lactobacillus*, *Bifidobacterium*, *Clostridium*, *Bacteroides* and *Enterococcus* (Song et al., 2019). Interestingly, 2,818 BSH-encoding genomes were found in wild rodents, many affiliated with *CAG-485* and *CAG-873* (*Muribaculaceae* and *Lachnospiraceae*, respectively), emphasizing their central role in modulating the host bile acid pool.

Microbes encoding 7α-HSDH, involved in the oxidation of deconjugated BAs, were less abundant but still critical (Funabashi et al., 2020). We identified 609 such genomes, primarily from *Bacteroidota* (Ferrandi et al., 2012). In contrast, only 34 genomes encoded *baiB*, a key gene in secondary BA synthesis (Mihalik et al., 2002), suggesting that late-stage BA transformation is limited to a small subset of taxa. These observations highlight the broad yet uneven distribution of BA metabolic capabilities and reinforce the ecological importance of these microbial pathways (Lin et al., 2023).

BA metabolism varies widely across host species due to differences in host physiology and environmental exposure (Thakare et al., 2018; Zhang et al., 2022). Comparative analyses revealed distinct patterns in BA metabolic gene profiles between wild rodents and other species (humans, pigs, chickens, and laboratory mice), reflecting co-evolution between microbial communities and host-specific bile acid compositions (Sinha et al., 2020; Sayin et al., 2013). For example, bacterial 7β-HSDHs mediate the conversion of 7-oxo-lithocholic acid to ursodeoxycholic acid, a bile acid with therapeutic applications (Lee et al., 2013; Ferrandi et al., 2012; Liu et al., 2011). Interestingly, the 7β-HSDH gene was absent in laboratory mice, highlighting divergences in BA pathways likely driven by domestication and constrained microbial diversity (Wei et al., 2020; Ma et al., 2020). While laboratory mice are foundational to gut microbiota research, their controlled environments may constrain microbial diversity and functionality (Hanski et al., 2024; Rosshart et al., 2017), limiting their suitability as models for BA-related studies. These findings advocate for the increased use of wild-type mice as more ecologically relevant models in microbiome and bile acid research.

Although dietary differences undoubtedly shape gut microbial communities, recent studies have demonstrated that host genotype and ecological context exert profound influences on microbial function independent of diet (Rothschild et al., 2018; Maurice et al., 2015). Moreover, research on wild versus captive animals has revealed consistent shifts in microbial diversity and metabolic potential due to domestication and environmental constraints (Clayton et al., 2016; Reese and Dunn, 2018). Wild rodents, living under natural ecological conditions and exposed to diverse microbial and dietary inputs, exhibit distinct BA-transforming microbial profiles compared to laboratory mice. These differences likely reflect long-term host-microbe co-adaptation and ecological pressures rather than dietary influences alone.

Bile tolerance is a vital trait that enables microbial communities to survive and function effectively within the intestinal environment (Lin et al., 2023). In this study, functional profiling of BSH-positive genomes, particularly from the dominant *CAG-485* lineage, revealed key metabolic traits that may confer bile resistance and competitive advantages. The enrichment of genomes in glycoside hydrolase (GH) families *GH13* and *GH16*, essential for carbohydrate metabolism, suggests that BSH-positive microbes possess an expanded capacity for energy acquisition and niche adaptation. Notably, *GH63* enzymes, capable of hydrolyzing α-glucosidic linkages in host-derived glycans and dietary polysaccharides (Kelly et al., 2016), were exclusive to BSH-positive taxa. Of particular interest, GH63’s ability to cleave α-linked L-arabinofuranosyl residues in plant hemicellulosic polysaccharides (Saito et al., 2020) may enhance microbial fitness in bile-rich environments, although its direct role in BA metabolism remains unclear. These findings highlight the metabolic versatility of BSH-positive microbes and illustrate a sophisticated interplay between carbohydrate and bile acid metabolism that supports microbial adaptation and functional dominance within the gut microbiome.

Parasite, such as helminths and protozoa, can also influence gut microbiome communities (Barash et al., 2017; McKenney et al., 2015; Aivelo and Norberg, 2018). In this study, *E. bieneusi* infection was associated with increased microbial diversity and elevated *Bacillota_C* abundance, suggesting infection-driven shifts in BA-relevant microbial taxa. Members of *Bacillota* are known to release immunomodulatory molecules such as peptidoglycan (Jordan et al., 2023), which may influence host immune responses. Additionally, infected individuals exhibited increased levels of *BSH*, *7α-HSDH*, and *baiB*, indicating a possible upregulation of BA transformation pathways during infection. These enzymatic changes could impact lipid metabolism and immune homeostasis (Guan et al., 2022; Wang et al., 2024a). Previous research has shown that elevated cholic acid can promote the growth of *Bacillota* species capable of 7α-dehydroxylation (Ridlon et al., 2013). Thus, parasitic infections may enhance microbial BA metabolism, with potential consequences for host immunity and susceptibility to secondary infections (Pickard et al., 2017; Collins et al., 2023). Taken together, our results indicate that *E. bieneusi* infection promotes a more metabolically active and immunomodulatory gut microbiota, possibly reshaping the intestinal environment to influence host physiological status.

Metabolic profiling of BAs using advanced detection technologies offers accurate and comprehensive monitoring of their composition and concentrations, which is essential for disease prevention, diagnosis, and treatment (Liu et al., 2018). It is important to note that our findings are based on genomic potential rather than direct biochemical measurements of bile acid species. While this approach enables comprehensive and high-resolution identification of microbial metabolic capabilities (Coelho et al., 2022; Paoli et al., 2022), it does not capture actual changes in bile acid concentrations or profiles. Future studies integrating metagenomics with metabolomics and host physiological data will be critical to fully elucidate the interplay between microsporidian infection, bile acid metabolism, and host health.

## 5 Conclusion

This study offers a comprehensive and functional perspective on the gut microbiota of wild rodents, shedding light on the specialized role of microbial communities in BA metabolism. The dominance of *Muribaculaceae*, particularly *CAG-485*, in BA transformation highlights an underappreciated microbial function with potential implications for host metabolism. Distinct host-specific patterns in BA metabolism were observed, with wild rodents exhibiting microbial adaptations that differ markedly from those of laboratory mice and other domesticated animals—suggesting that standard model organisms may not fully capture the complexity of natural gut microbiomes. Furthermore, infection with *E. bieneusi* significantly altered the composition of BA-associated microbes, pointing to the dynamic responsiveness of gut microbiota to pathogen colonization. BSH-positive microbes demonstrated clear functional adaptations, including enriched glycoside hydrolase activity, supporting enhanced metabolic efficiency and survival in the bile-rich intestinal environment. These findings underscore the intricate relationship between gut microbes and host physiology, emphasizing the ecological and metabolic flexibility of the wild rodent microbiome. Future studies should aim to experimentally validate these microbial pathways and explore their influence on host health, immunity, and resistance to disease.

## Data Availability

The datasets presented in this study can be found in online repositories. The names of the repository/repositories and accession number(s) can be found in the article/**Supplementary Material**.

## Supplementary material

Supplementary Figure 1 Bile acid transformation pathways in 5,208 genomes of intestinal microorganisms from wild rodents. **(A)** Deconjugation of glycocholic acid (GCA) and taurocholic acid (TCA), followed by conversion to 7-ketocholic acid via 7α-hydroxysteroid dehydrogenase (7α-HSDH) or to cholyl-CoA via *baiB*. **(B)** Deconjugation of glycochenodeoxycholic acid (GCDCA) and taurochenodeoxycholic acid (TCDCA), leading to the formation of 7-dehydro-chenodeoxycholic acid. **(C)** Conversion of chenodeoxycholic acid (CDCA) to CDCA-CoA via the *bai* pathway. **(D)** Conversion of ursodeoxycholic acid (UDCA) to UDCA-CoA via the *bai* pathway.

Supplementary Figure 2 Genus-level classification of BSH-carrying MAGs from the intestinal microbiomes of wild rodents (wild), humans, pigs, laboratory mice (Lab), and chickens.
