# Supplementary figures and images for: Enterocytozoon bieneusi infection disrupts bile acid metabolism in the wild rodent gut microbiota: adaptive shifts in microbial metabolism and community structure
Source: Front Cell Infect Microbiol. 2025 Sep 22;15:1647377. doi: 10.3389/fcimb.2025.1647377 (PMC12497767; doi:10.3389/fcimb.2025.1647377)

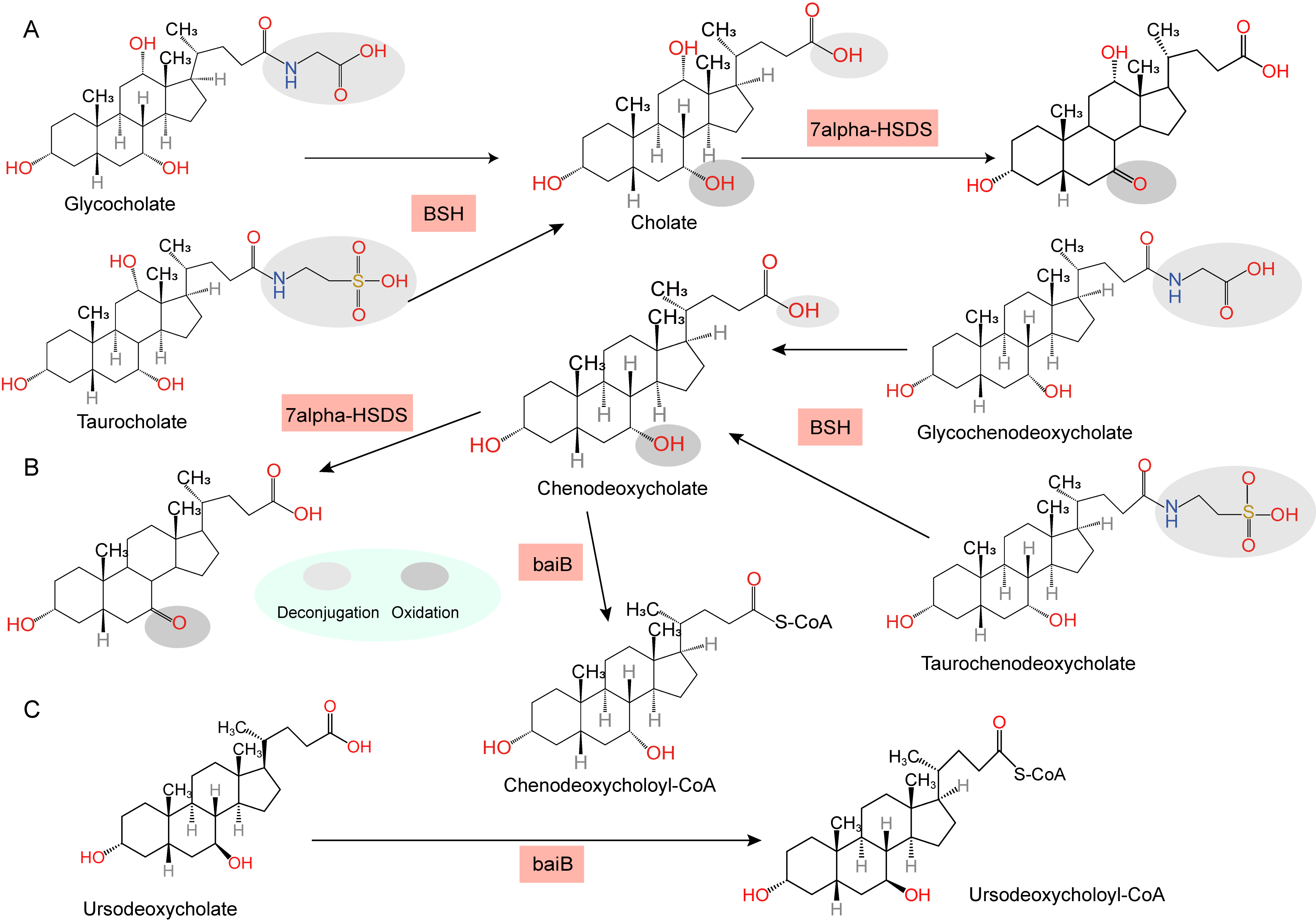

Supplement: Supplementary Figure 1 — Bile acid transformation pathways in 5,208 genomes of intestinal microorganisms from wild rodents. (A) Deconjugation of glycocholic acid (GCA) and taurocholic acid (TCA), followed by conversion to 7-ketocholic acid via 7α-hydroxysteroid dehydrogenase (7α-HSDH) or to cholyl-CoA via baiB. (B) Deconjugation of glycochenodeoxycholic acid (GCDCA) and taurochenodeoxycholic acid (TCDCA), leading to the formation of 7-dehydro-chenodeoxycholic acid. (C) Conversion of chenodeoxycholic acid (CDCA) to CDCA-CoA via the bai pathway. (D) Conversion of ursodeoxycholic acid (UDCA) to UDCA-CoA via the bai pathway. [file Image1.tif]

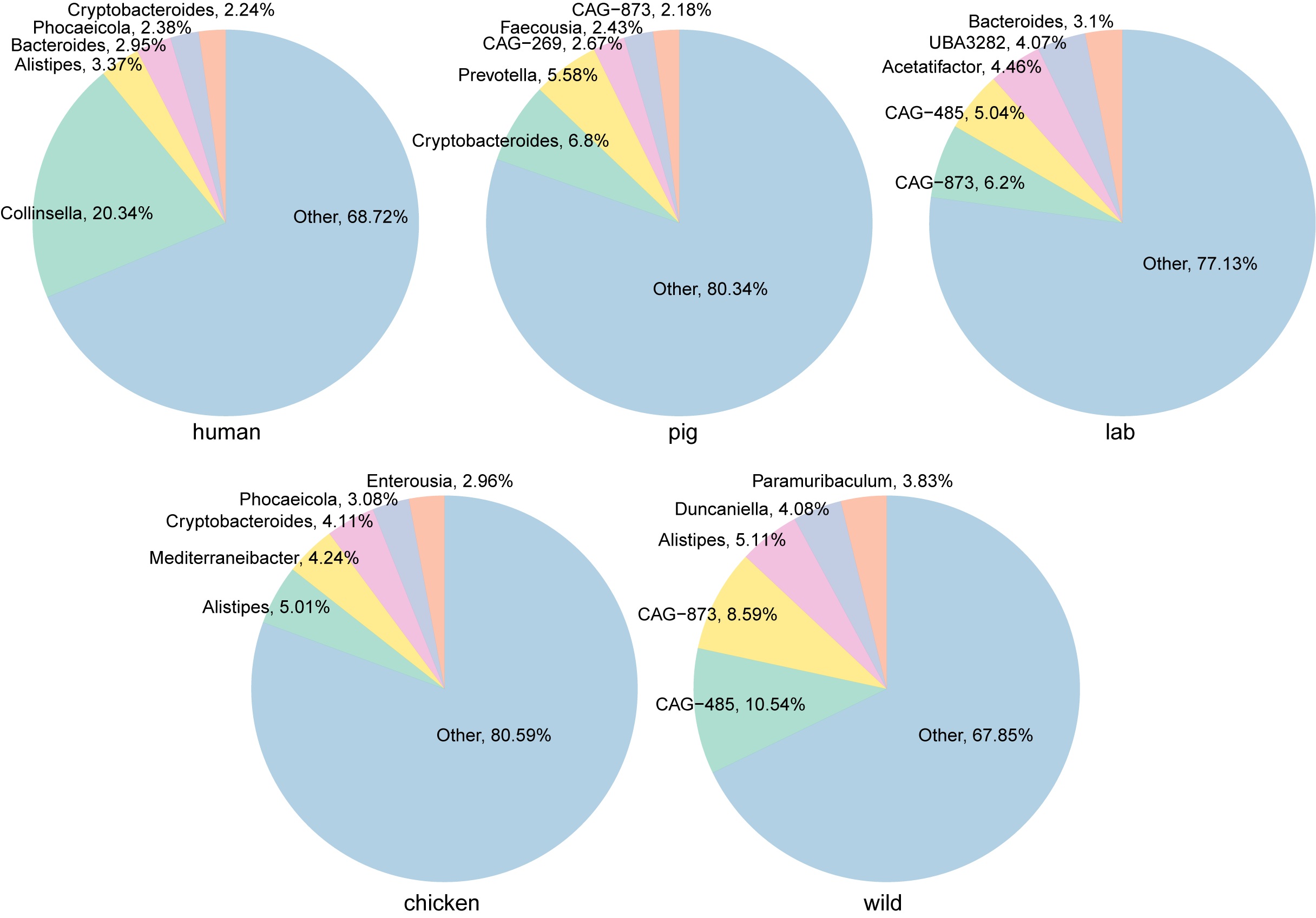

Supplement: Supplementary Figure 2 — Genus-level classification of BSH-carrying MAGs from the intestinal microbiomes of wild rodents (wild), humans, pigs, laboratory mice (Lab), and chickens. [file Image2.tif]
